# Hemophagocytic syndrome related by EBV infection: case report

**DOI:** 10.1093/omcr/omae045

**Published:** 2024-05-20

**Authors:** Maria Cristina De Santis, Elisa Martinelli, Anna Lo Cricchio, Paolo Mercatelli, Giulia Campanaro, Alessandra D’Arienzo, Alberto Moggi Pignone, Giulia Bandini

**Affiliations:** Department of Experimental and Clinical Medicine, Division of Internal Medicine, University of Florence, Florence, Italy; Department of Experimental and Clinical Medicine, Division of Internal Medicine, University of Florence, Florence, Italy; Department of Experimental and Clinical Medicine, Division of Internal Medicine, University of Florence, Florence, Italy; Department of Experimental and Clinical Medicine, Division of Internal Medicine, University of Florence, Florence, Italy; Department of Experimental and Clinical Medicine, Division of Internal Medicine, University of Florence, Florence, Italy; Department of Experimental and Clinical Medicine, Division of Internal Medicine, University of Florence, Florence, Italy; Department of Experimental and Clinical Medicine, Division of Internal Medicine, University of Florence, Florence, Italy; Department of Experimental and Clinical Medicine, Division of Internal Medicine, University of Florence, Florence, Italy

## Abstract

A 34-year-old woman of Asian origin with diffuse lymphadenopathy and hepatosplenomegaly in hemophagocytic syndrome induced by Epstein Barr Virus (EBV) infection. The rapidity of progression of clinical manifestations lead to early orotracheal intubation and death due to multiple organ failure (MOF).

## INTRODUCTION

**Figure 1:**
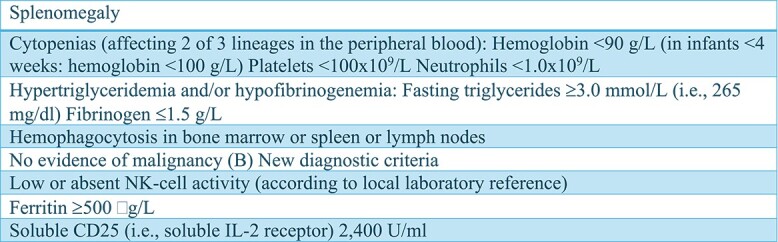
HLH-2004 Criteria.

Hemophagocytic lymphohistiocytosis (HLH) is a rare condition that causes a massive activation of the immune system due to a dysregulation of cytolytic secretory pathway of T and NK cells resulting in a cytokine storm. It is a potentially mortal condition and death is due to multiple organ failure. It is classified in primary and secondary forms: the former is associated with genetic defects, while the latter is a condition in which a trigger (e.g. an infection or a tumor) activates the disease. Epstein–Barr virus (EBV) is the most frequent infection associated with HLH [1]. One of the main diagnostic criteria are HLH-2004 [2] (Fig. 1) but in clinical practice the probability score Hscore is also used [2]. Even if this condition is extremely rare, the outcome can be fatal but early detection can be life-saving; this was the main reason behind our decision to describe this case report. Our patient presented all typical clinical features of fulminating HLH variant and unfortunately the outcome could not be avoided despite the therapy.

## CASE REPORT

### Patient’s presentation

A 34-year-old Chinese woman arrived on our ward. She had no underlying comorbidities, however, she had developed lymphadenopathies in the neck approximately two months after the second dose of SARS-CoV2 vaccine. Prior to admission she had undergone neck and abdomen ultrasound (US) and a neck-chest-abdomen computed tomography (CT scan) which showed cervical, submandibular, supraclavicular, axillary, left internal mammary chain, right mediastinal, periaortic, intercavoaortic, inguinal lymphadenopathies. The CT scan also showed hepatomegaly without local lesions and splenomegaly (diameter 17 cm) with accessory splenic nodules. Blood tests showed leukopenia, thrombocytopenia, anemia, and an increase of erythrocyte sedimentation rate (ESR).

### Clinical

On physical examination the patient presented a fever. There were bilateral palpable nodes in the neck, the largest were at submandibular and at the axillary and inguinal level, on the right side. On abdominal evaluation, the hepatic margin was regular but palpable 4 cm from the costal arch; the splenic margin was also palpable 3 cm from the costal arch.

### Diagnostic exams

**Figure 2:**
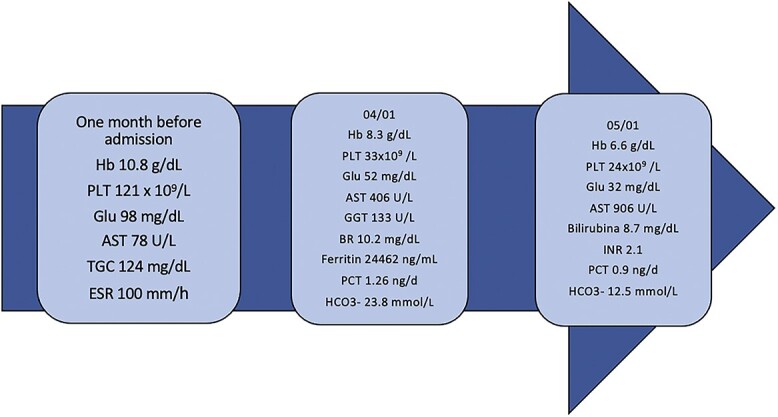
Time-line of blood alteration. AST: Aspartate Transaminase, BR: bilirubin, ESR: erythrocyte sedimentation rate, GGT: gamma glutamyl transferase, Glu: glucose, Hb: hemoglobin, PLT: platelets, INR: International Normalized Ratio, PCT: procalcitonina, TG: triglycerides.

The alterations in blood count previously found were confirmed and there was severe thrombocytopenia (33×10^9^/L), hemolytic anemia, increased liver enzymes, increased gamma-GT and alkaline phosphatase, hypoglycemia and hyponatremia (Fig. 2).

**Figure 3:**
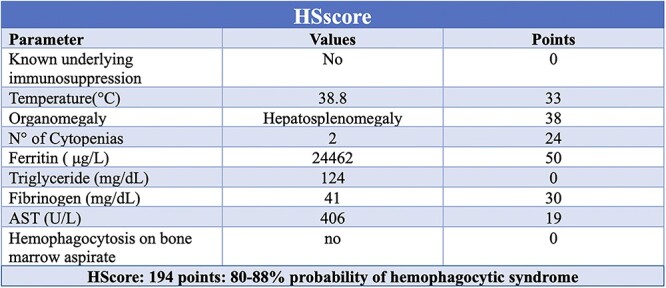
Patient’s HScore.

The patient also presented increased inflammatory markers. Due to the recent neck chest and abdomen CT, no further instrumental exams were performed. From a microbiological point of view, more than one million copies of EBV DNA were detected in the patient bloodwork, confirming an ongoing viral infection. We performed the HScore with a 80%–88% probability of hemophagocytic syndrome (Fig. 3). According to the HLH-2004 criteria the patient had five out eight criteria (Splenomegaly, Fever, Cytopenia affecting 2 of 3 lineages in the peripheral blood with anemia and thrombocytopenia, Ferritin ≥500 g/L, Fibrinogen 1.5 ≤ g/L), hence the diagnosis of HLH.

### Treatment

As indicated by our colleagues of the Hematology Department, we started treatment with dexamethasone 40 mg/die and performed a bone marrow biopsy. The procedure was unsuccessful, and it was not possible to repeat the procedure due to the patient’s poor compliance. It was followed by a sudden worsening of the patient’s clinical conditions characterized by hypoglycemia, tachypnea, tachycardia, abdominal distension, spontaneous gingival and nasal bleeding. The patient was transferred, due to her critical conditions, to an intensive care unit where she continued to deteriorate. A severe lactic acidosis associated with severe hypoglycemia (32 mg/dL, not responsive to administration of Glucose Solution 33%) and severe hyponatremia were found. Fibrinogen and a concentration of coagulation factor (II, VII, IX, X, protein C and S) were initially administrated. The patient underwent transfusions of fresh frozen plasma, red blood cell concentrates and platelets. Respiratory function progressively worsened leading to endotracheal intubation and hemodynamic stability was maintained by aminic support. The patient died approximately 24 h after hospitalization. Death was due to multiple organ failure.

## DISCUSSION

The epidemiological characteristics of the case report agree with the literature on hemophagocytic syndrome: an Asian patient with EBV infection, which is more commonly found in population from Asian countries [3]. The HScore and the HLH-2004 criteria were very important to detect this condition and to allow the administration of intravenous steroids, first line therapy according to the most recent therapeutic protocols [2]. In literature we couldn’t find other case report of EVB-related HLH, however there are some cases of fulminant variants due to CMV, HAV, Lymphoma [4] and pandemic A Influenza (H1N1) [5]. All patient shortly died despite the therapy and in three cases the outcome was due to multiple organ failure. The main clinical feature presented by all patients are fever, hepatosplenomegaly, thrombocytopenia. Even though in our case about HLH was a secondary form due to an infection, it was still impossible to administer an eradicating therapy aimed at removing the trigger and reducing systemic inflammation, since there is no target therapy of Epstein Barr Virus. The patient was a candidate for therapy with etoposide as a rescue therapy [1] but it was not administered because of early passing. On the other hand, EBV infection represents a negative prognostic factor [3] and the syndrome has an in-hospital mortality rate of 20%–75% [6, 7].

## CONCLUSION

Hemophagocytic syndrome remains a challenge for clinicians, as it is a rare manifestation that occurs in association with the most diverse medical condition. The rapid and sudden onset and its systemic clinical consequences increase the likelihood that patients will require a higher level of care in regular wards and intensive care units.

## CONFLICT OF INTEREST STATEMENT

The authors have no competing interest to declare that are relevant to the content of this article.

## FUNDING

There are no sources of funding.

## ETHICAL APPROVAL

No approval was required. The study was conducted following Good Clinical Practice recommendations, in accordance with Helsinki declaration. All authors complied with the ethics and policy of the journal.

## GUARANTOR

Prof. Alberto Moggi Pignone, A. Moggi Pignone, reparto di Medicina Interna 4, AOU-Careggi, Firenze, 50 134, Italia, 0557947340, alberto.moggipignone@unifi.it

## DATA AVAILABILITY

Data on this case report is available from the authors upon reasonable request.
